# How Can Dolphins Recognize Fish According to Their Echoes? A Statistical Analysis of Fish Echoes

**DOI:** 10.1371/journal.pone.0014054

**Published:** 2010-11-19

**Authors:** Yossi Yovel, Whitlow W. L. Au

**Affiliations:** 1 Department of Zoology, Tel-Aviv University, Tel-Aviv, Israel; 2 Marine Mammal Research Program, Hawaii Institute of Marine Biology, University of Hawaii, Kailua, Hawaii, United States of America; University College London, United Kingdom

## Abstract

Echo-based object classification is a fundamental task of animals that use a biosonar system. Dolphins and porpoises should be able to rely on echoes to discriminate a predator from a prey or to select a desired prey from an undesired object. Many studies have shown that dolphins and porpoises can discriminate between objects according to their echoes. All of these studies however, used unnatural objects that can be easily characterized in human terminologies (e.g., metallic spheres, disks, cylinders). In this work, we collected real fish echoes from many angles of acquisition using a sonar system that mimics the emission properties of dolphins and porpoises. We then tested two alternative statistical approaches in classifying these echoes. Our results suggest that fish species can be classified according to echoes returning from porpoise- and dolphin-like signals. These results suggest how dolphins and porpoises can classify fish based on their echoes and provide some insight as to which features might enable the classification.

## Introduction

Various experiments have shown that dolphins and porpoises can perform complex biosonar target discrimination tasks [1]. The objects used in these experiments were always foreign to dolphins and porpoises but familiar to humans (e.g., metallic spheres, disks, cylinders). These experiments have provided much knowledge about the target discrimination and recognition capabilities of the dolphin biosonar system, yet we gained only little insights on how dolphins and porpoises can forage for prey in the wild. It is extremely difficult to address the issue of selective foraging by dolphin and porpoise because of the difficulties in making good, regular and consistent observations of underwater foraging behavior in the wild. Despite this however, recent studies provide more and more evidence for prey selection by cetaceans [2], [3], [4]. One of the clearest cases of selective foraging has been described for fish eating killer whales in the waters of British Columbia where even in months when Chinook salmon may constitute less than 15% of the salmon population; the whales still forage mainly on Chinook salmon [4]. Visual observations of foraging killer whales strongly suggest that they depend on echolocation to detect and recognize their prey: Whales would often be observed swimming near the surface along nearly straight line tracks for minutes and then suddenly submerge and resurface several tens of meters away with a salmon in their mouths. Collection of scales after the whales bring the prey to the surface have allowed for the identification of the salmon species.

In order to learn more about the possibility to recognize fish according to its echo, Au et al. [5] measured the acoustic backscatter from four species of fish, Atlantic cod, Mullet, Sea bass and Pollack using simulated dolphin and porpoise biosonar signals. In this work we shall introduce a two types of classifiers to test if and how might dolphin and porpoise use their biosonar system to select specific prey.

## Results

**Figure 1:**
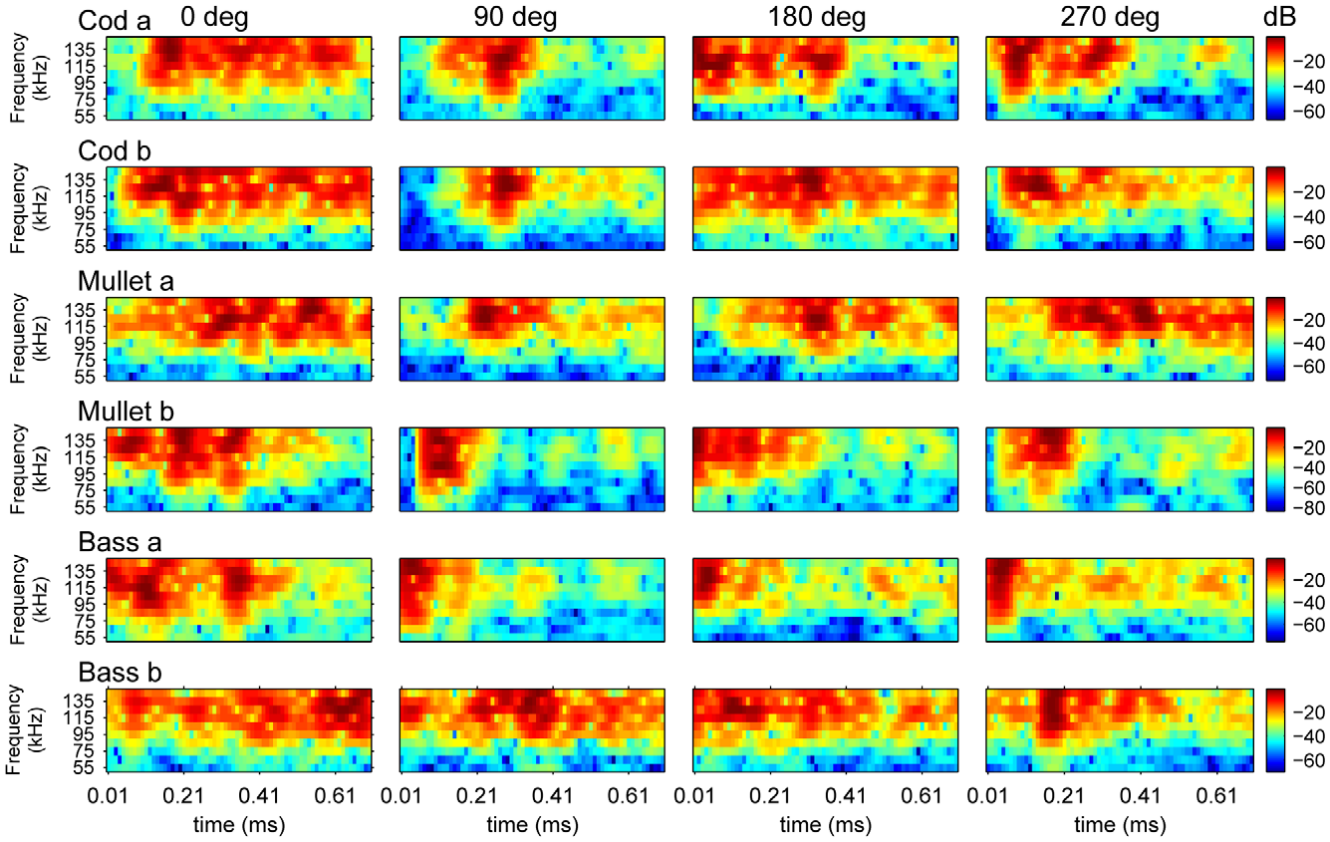
Fish echoes are characterized by high variability. Raw magnitude spectrograms of two specimens of each of the three fish species used in the experiments generated with a dolphin signal, each from four acquisition angles. Color code is in dB.

Fish echoes are characterized by large intra-species variability and a strong dependency on the angle of acquisition (Figure 1).

### Statistical classification using a simple parametric approach

We started with a simple parametric approach that relied on the extraction of six statistics [6] from the envelopes of the time series of the fish echoes and the application of discriminant function analysis (DFA) to classify them (see Materials and Methods). The purpose of this parametric approach was not to show how dolphins can classify fish echoes, but only to test whether such a simple classification strategy would reveal species-specific cues that might suggest that classification is possible. Therefore, we do not argue that this approach and the extracted parameters are biologically plausible and available to the animals. The reason for this pre-test was our limited sized data set: the finding that a simple approach like this reveals species-specific cues (see below) was a strong confirmation that the features found by our more complex machine-learning-based algorithm might be real.

Classification based on a simple parametric approach strongly suggests that species specific cues are available in the echoes. We could probably increase the performance by calculating the impulse response of the fish (by cross correlating the echo with the emitted signal) and thus emphasizing fine temporal details. However, we preferred not to do so and to rely on a lower resolution representation of the data that is more similar to the data used by the described below (machine-learning based) approach.

#### Dolphin signals

In almost all cases, the DFA-based classifier was able to classify the tested echoes with performance significantly higher than chance (t-test, Table 1). The task of classifying Cod seemed to be easier than the other two, while the classification of Sea bass seemed to be the most difficult and statistical results for its classification were not better than chance for echoes from the tail aspect (see Materials and Methods) and from all aspects. In general, the classification of echoes from one aspect, especially the tail aspect improves the performance in comparison to the usage of echoes from all angles. However, for at least Cod and Mullet, it seems that some features are general and enable classification invariantly of the aspect angle. We performed a post-hoc analysis to determine which of the statistics extracted from the echoes is more informative (i.e., more important for classification) by repeatedly running the classifiers after excluding each of the statistics. The results of this analysis are somewhat ambiguous, suggesting that different parameters are important for the classification of different species. The crest factor (the ratio between the peak amplitude and the root mean square) and the second moment for instance (see Materials and Methods) seem to be less important for Sea bass classification on the one hand, but very important for Mullet classification on the other hand.

**Table 1:** DFA classification performance of fish echoes created with a dolphin-like signal.

Cod (%)			Mullet (%)			Sea Bass (%)		
All angles	Tail aspect	Broadside aspect	All angles	Tail aspect	Broadside aspect	All angles	Tail aspect	Broadside aspect
68±13*	72±14*	65±14*	49±16*	53±17*	50±15*	34±15	25±18	44±14*

Asterisk – significantly above chance level (P<0.05 chance level = 33%).

#### Porpoise signal

Classification performance based on echoes generated with a porpoise-like signal was somewhat reduced in comparison to the dolphin signal (t-test, Table 2). Sea bass was actually inseparable using this method and so was cod only from the broadside aspect. This reduced performance could be a result of the lesser resolution of fine temporal details in the porpoise echoes dictated by the longer pulse duration and the narrower. For both the porpoise and dolphin signals, the classifier tended to mistake Sea bass echoes mainly (but not only) with Mullet. Still, the general finding that species specific information is available in the echoes holds for the porpoise echoes as well.

**Table 2:** DFA classification performance of fish echoes created with a porpoise-like signal.

Cod (%)			Mullet (%)			Sea Bass (%)		
All angles	Tail aspect	Broadside aspect	All angles	Tail aspect	Broadside aspect	All angles	Tail aspect	Broadside aspect
50±14*	63±17*	33±15	57±17*	40±13*	68±17*	33±14	26±14	28±13

Asterisk – significantly above chance level (P<0.05, chance level = 33%).

### Classification using a Support Vector Machines (SVM)

The Support Vector Machines used by us are linear classifiers that seek a decision rule that is based on a linear combination of features extracted from the raw spectrograms of the echoes. The rather low spectral-temporal resolution of the magnitude-spectrograms we used along with the fact that they did not contain any phase information certify that this classification approach is biological plausible in the sense that the information it bases classification on is available to the mammalian auditory system (see Materials and Methods). We therefore preferred to show that classification is possible with a low-resolution representation of the data, assuming that any higher resolution will only improve classification.

#### Dolphin signal

Despite the echoes' high intra-species variability and their strong dependency on the angle of ensonification, fish species can be classified with high accuracy based on a single echo's spectrogram from any angle. Classification was significantly above chance level both for the pair-wise classification tasks (i.e., each species vs. any of the other two) and for the one vs. all classification task (Table 3). As expected, the classifiers performed better for the pair-wise classification task. Both types of error (i.e., true negatives and false positives) were more or less equal. Like in the case of the DFA classifiers, the task of Mullet vs. Sea Bass was found to be the most difficult. Because of the very high classification performance for the case of data from all aspect angles, we did not test the case of using only part of the angles (i.e., only tail aspect or only broadside aspect).

**Table 3:** SVM classification performance of fish echoes created with a dolphin-like signal.

Cod vs. Mullet (%)		Mullet vs. Sea Bass (%)		Sea Bass vs. Cod (%)	
Raw	Smoothed	Raw	Smoothed	Raw	Smoothed
96±1*	92±3*	96±1*	82±3*	97±1*	95±2*

Cod vs. The Rest (%)		Mullet vs. The Rest (%)		Sea Bass vs. The Rest (%)	
Raw	Smoothed	Raw	Smoothed	Raw	Smoothed
91±1*	85±3*	85±2*	65±9*	92±1*	78±5*

Asterisk – significantly above chance level (p<0.05, chance level is 50%).

#### The decision echoes

**Figure 2:**
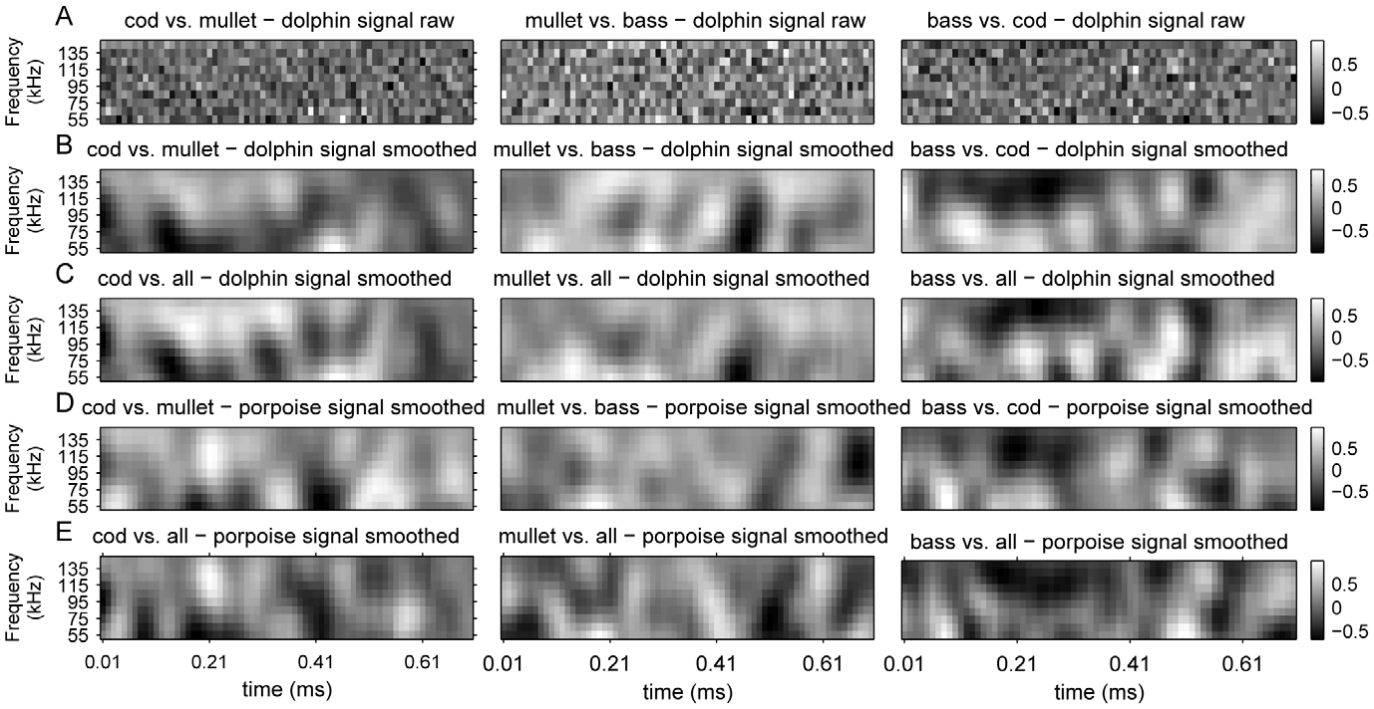
Graphical representation of the decision echoes learned by the SVMs. Each decision echo corresponds to a weight vector (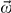). The decision about the class (species) of the fish is reached according to the sign of the inner product of the weight vector with the fish's spectrogram. (A) Decision echoes of classifiers trained with echoes from a dolphin signal and no smoothing. (B) Smoothed decision echoes of classifier trained with echoes generated with dolphin signals for pair-wise classification tasks. (C) Smoothed decision echoes of classifier trained with echoes generated with dolphin signals for one against all classification tasks. (D) Smoothed decision echoes of classifier trained with echoes generated with porpoise signals for pair-wise classification tasks. (E) Smoothed decision echoes of classifier trained with echoes generated with dolphin signals for one against all classification tasks.

The weight vector (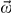) that is learned by the linear classifier (SVM), which we term the decision echo, represents the decision rule learned by the classifier (see Materials and Methods). The regions of the decision echo that have high absolute values are more important for classification. An examination of the decision echoes can thus tell us if the classifier learned meaningful features of the data or simply used whatever it could find. The latter implies that the data might be linearly separable only because it is a small subsample of the full world. The raw decision echoes learned by the classifier (Figure 2A) are very noisy (contain a lot of high frequencies) and are therefore likely to contain artifacts that are a result of the small sample-size.

The smoothed decision echoes however (see Materials and Methods) still enable highly significant classification performance (t-test, Table 3) and reveal salient structures that could imply a meaningful decision rules. These rules, as appear in the smoothed decision echoes, seem to contain both temporal and spectral features which cover the entire spectral-temporal range of the echoes (Figure 2B&C). Interestingly, many of the features that appear in the decision rule for the pair-wise situation are also apparent in the one vs. all situations.

#### Porpoise signal

As in the case of the dolphin signal, fish species can be classified with high accuracy based on a single echo's spectrogram created by a porpoise signal from any angle. This is true both in the pair classification task and in the one vs. all tasks (Table 4). As in the dolphin case, the smoothed decision echoes of these classifiers perform very well (t-test, Table 4) and show clear structures that seem to contain both temporal and spectral cues which cover the entire spectral-temporal range of the echo (Figure 2D&E).

**Table 4:** SVM classification performance of fish echoes created with a porpoise-like signal.

Cod vs. Mullet (%)		Mullet vs. Sea Bass (%)		Sea Bass vs. Cod (%)	
Raw	Smoothed	Raw	Smoothed	Raw	Smoothed
98±1*	72±6*	98±1*	89±6*	97±1*	79±4*

Cod vs. The Rest (%)		Mullet vs. The Rest (%)		Sea Bass vs. The Rest (%)	
Raw	Smoothed	Raw	Smoothed	Raw	Smoothed
92±1*	75±4*	92±1*	83±5*	92±1*	73±5*

Asterisk – significantly above chance level (P<0.05, chance level is 50%).

Similar to the case of DFA classifiers, the performance here (for the smoothed decision echoes) was not as good as that achieved by using a dolphin signal. Interestingly, Cod which was easy to classify in all other cases (i.e., DFAs and SVMs with dolphin-like signal), was rather difficult in this case.

#### Validation

All three validation methods that we used (i.e., smoothing of the decision echoes, resampling the data using a principle component analysis and testing resistance of results to noise, see Materials and Methods for details and Tables 5 and 6) revealed that the decision rules found by the SVM classifier are robust and resistant to noise. The results for the smoothed decision echoes are given in Tables 3 and 4. The Results for the noise sensitivity test are given in Tables 5 and 6 and the performance for the principle component analysis resampled data were still high above change level in most cases (last row in Tables 5 and 6). These validation methods increase our confidence that the results described above do not originate from some artifact that is a result of the small sample size of the data and might be general for larger, closer to reality, data sets.

**Table 5:** Sensitivity of dolphin classifiers to noise.

Average Noise (% from max intensity)	Cod vs. The Rest (%)	Mullet vs. The Rest (%)	Sea Bass vs. The Rest (%)
10	85±3	55±10	60±3
20	86±3	52±6	64±7
30	85±3	56±15	64±5
40	83±4	53±12	62±8
50	82±4	55±11	67±6
PCA resampled data	81±2	61±14	59±2

This was tested only for the one vs. all classification tasks.

**Table 6:** Sensitivity of porpoise classifiers to noise.

Average Noise (% from max intensity)	Cod vs. The Rest (%)	Mullet vs. The Rest (%)	Sea Bass vs. The Rest (%)
10	75±4	82±3	66±7
20	77±3	82±4	68±9
30	74±6	74±3	71±2
40	75±4	78±4	67±10
50	71±11	75±7	61±10
PCA resampled data	78±7	67±4	53±5

This was tested only for the one vs. all classification tasks.

## Discussion

Au et al. [5] introduced the use of natural prey echoes to the study of echo based object classification by dolphins and porpoises. Here we take their work one step forward by suggesting a machine learning based linear classifier that can deal with the high-dimensional data representing a fish's echo and provides insight on if and how might potential prey be classified according to its echo.

The linear classifiers we used (namely, SVMs) learned to classify each of the three fish species we tested with accuracy high above chance level based on a single spectrogram invariantly of the angle of acquisition. The smoothed decision echoes (Figure 2) depicting the rules learned by the classifier, are characterized by clear hyper and hypo-intensity blob-like structures that represent areas in the spectral-temporal representation of the echoes that are important for classification. These structures might correspond to the size and shape of the fish's swimming balder which result in reflection returning at certain time instants and with specific frequency response as well as to different multi-path reverberation patterns. The blobs seem to appear along the entire echo and cover the entire spectral range of the echo.

Due to the limited sample size of our data, our results are not sufficient to prove that echo-based classification of fish species is possible using SVMs. The very high performance and the salient features that were found beneficial for classification however, suggest that the approach presented here might be relevant also for dealing with the variability in the real world. In addition, the fact that DFAs relying on only six parameters were able in most cases to perform significantly above chance level, strongly suggests that species-specific cues are available in the echoes. The assumptions made by our method regarding the temporal and spectral resolution needed for classification are minimal and thus assure that data we use is available to the animal. Also, we only tested classification based on a single echo, while using several echoes from several aspects should improve classification. In order to increase the confidence in these results, more echoes from many more specimens should be collected.

### Biological plausibility

The machine learning based classification approach described in this work could be easily applied by dolphins and porpoises. The temporal and spectral resolution of the data extracted from the spectrograms is probably plausible for the dolphin auditory system [7] and should thus not be a limiting factor. Moreover, the high performance of the smoothed decision echoes (see Results) implies that the temporal resolution can be reduced without harming classification much. The decision rule applied by the SVM classifier is biologically plausible in the sense that it can be implemented by a network of neurons with spectro-temporal receptive fields that match the features extracted by the SVM (Figure 2). Recent studies have even found neurons in the Ferret auditory cortex that have spectral-temporal filters that resemble in their shape, (and not their absolute temporal-spatial resolution) the features extracted by the SVM classifier [8] suggesting that such features might be encoded in the auditory cortex. Moreover, the decision rule of a SVM classifier was recently shown to correlate with the behavior of greater mouse-eared bats performing an acoustic classification task, although the classifiers that were found to behave most similarly to the bats' behavior were non-linear SVMs [9]. However, despite the above, we cannot argue about the similarity of our method and the animals' behavior. Only carful behavioral experiments that compare the statistical classifiers to the animals' behavior will be able to determine the similarity of the results of our approach to the animals' behavior.

### Dolphin signals vs. Porpoise signals

Both classification methods we used suggested that the on average dolphin-like signals provide better classification performance than porpoise-like signals. The reason for this could be the shorter duration of the dolphin signal and its wider bandwidth. Because we did not use a matched-filter approach (and thus did not cross correlate the received echo with the emitted signal) the temporal information conveyed by the spectrograms we analyzed, are convolutions of the emitted signal and the impulse response of the fish. The shorter signal will thus “sharpen” the temporal information and reveal the temporal difference between the fish species. The wider band width might convey richer temporal details as revealed by interference patterns created by echoes returning nearby surfaces. In the case that the dolphin brain applies any sort of a correlation (coherent or non-coherent) process the wider band width would also assist in sharpening the temporal details of the processed echo. The wider band width also reveals more differences in the frequency response of the fish. The importance of the band-width for both of these aspects was suggested to play a role in discrimination tasks in echolocating bats [10], [11], [12].

Real dolphin signals tend to be even briefer than the one we used that was limited by the properties of the transducer. Following the explanation given above regarding the advantages of brief signals, real dolphin signals are expected to yield an even better performance than that achieved with our dolphin-like signal. Moreover, our results imply that due to the species specificity of the echoes, a wide range of signals will probably be adequate to classify fish species. The different signals might however be advantageous for classifying specific prey: In our case, cod was classified better with dolphin-like signals while mullet was easier to classify with the porpoise-like signal. This could be explained as a result of the signal's spectrum better emphasizing the frequency response of the object. The DFA analysis revealed that different prey can be best classified relying on different statistics measured from the echoes. For instance in our case, the crest factor and the second moment were less important for Sea bass classification, but very important for Mullet classification.

### Future analysis

At this point it is hard to connect the features learned by the algorithm as they are represented in the decision echoes, to the physical characteristics of the fish. In order to do so, one would need to acquire some decent representation of the physical structure of the fish (e.g. by using x-rays). Once such representations are available, the classification method suggested here, provides a powerful tool to connect the actual structure of the object to the features that are advantageous for classification according to the algorithm [13].

Another interesting approach would be to analyze the statistics of echoes returning from a school of fish (which can be thought of as an array of reflectors). Dolphins and porpoises could rely on the statistics of the school itself (e.g., typical distances within the school), similar to the approach suggested by Yovel et al. [14] to classify plants which are also arrays of reflectors with typical statistics.

Behavior studies that show dolphins and porpoises' ability to classify prey are needed to complete the story. However, such behavior studies would be difficult to conduct except perhaps under very restrictive conditions in which a phantom echo generator is used to generate fish echoes at different aspects [15], [16]. Humans acting as a proxy for dolphins are being used in a listening experiment to determine how well a mammalian auditory system can discriminated these fish echoes. Because of the inherent difficulties in performing dolphin biosonar experiments on fish discrimination classification algorithms such as the one introduced here can be used to lay a strong framework for modeling behavior. Using such classifiers, a hypothetic decision rule of the specific animal can be computed [9] and this, could later lead to performance of highly controlled behavioral experiments to test this rule.

## Materials and Methods

### Ethical Statement

This project complied with the Dutch standards for animal experiments (Chris Pool, Head of the Committee for Animal Experiments of RIKZ) and was conducted under University of Hawaii Animal Care Protocol 04-019. This is HIMB Contribution No. 1343.

### Experimental Geometry

**Figure 3:**
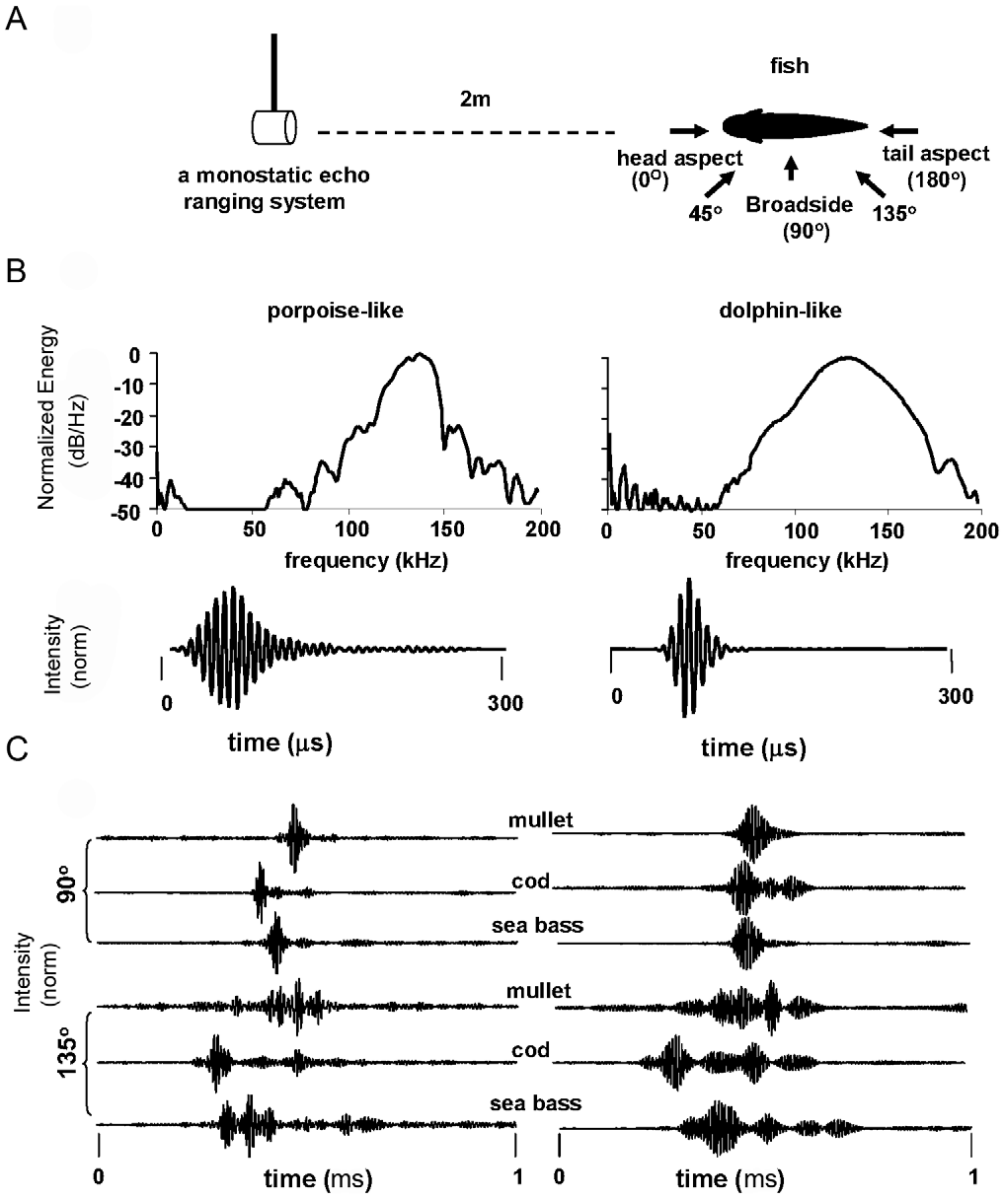
The experimental setting (adopted from reference 4). (A) Left: a monofilament net curtain attached to a rotor with a fish attached to the curtain. A monostatic echo ranging system was used to emit the signal and received the echoes. Right: The orientation system used in this study showing the direction of the incident signal with respect to the fish body. The two acquisition sections (tail aspect and broadside aspect) are indicated. (B) The simulated echolocation signals used in the study. Left: time signals and Right: spectra of the porspoise and dolphin signals. These signals were recorded using an acoustic mirror consisting of a flat 0.63 thick aluminum plate. (C) Examples of backscatter at different aspect angles for three of the fish species (a) with dolphin-like signal, (b) with the porpoise–like signal.

The backscatter measurements were conducted in an outdoor tank belonging to the Sea Mammal Research Company (Seamarco), at the field station of the Netherland's National Institute for Coastal and Marine Management (RIKZ) in Jacobahaven, Zeeland, The Netherlands. The surface dimension of the tank was 7 m×4 m with a water depth of 2 m. Anesthetized fish subjects were constrained in a monofilament bag that was in turn attached to a monofilament net which was attached to a rotor (Figure 3A).

### Fish Subjects

We examined three individuals of each of the following fish species: atlantic cod (*Gadus morhua*, length of 29 to 30 cm), grey mullet (*Chelon labrosus*, length of 15–17 cm) and sea bass (*Dicentrarchus labras*, length of 14–17 cm). All fish were on loan from “The Arsenaal Aquarium,” Vlissingen, The Netherlands. They were fed to satiation each day after the measurement sessions on a diet of raw fish and in compliance with The Animal Welfare Commission of The Netherlands. After the measurements they were returned to the aquarium. Since the fish were borrowed, we did not attempt to x-ray them and risk potential injury.

### Data Acquisition

The fish were rotated as simulated biosonar signals of *a* dolphin-like and porpoise-like were projected and the echoes collected (Figure 3A). Approximately 145 pings were emitted during one 360 rotation of the fish thus providing angular spacing of ∼2.5° between adjacent echoes. We define two alternative acquisition sectors (Figure 3A): 1) The tail aspect includes all ensonifications along the head-tail axis (from both sides) and up to 45 degrees away from it. 2) The broadside aspect is comprised of the rest of the acquisition angles. A monostatic system with the same transducer projecting the signals and receiving the echoes was used. The echoes were time-gated and filtered before being digitized at a sample rate of 1 MHz. A total of 1024 points were digitized per echo and stored to disk. The dolphin-like signal had a peak frequency of 130 kHz while the porpoise-like signal had a peak frequency of 138 kHz. The duration of the dolphin-like signal was approximately 70 µs versus 270 µs for the porpoise-like signal. The spectra of both signals are centered around 120–140 kHz, but the bandwidth of the porpoise signal was clearly narrower than that of the dolphin-like signal (Figure 3B). The dolphin-like signal had a duration and spectrum that resemble dolphin signals, but due to the properties of the emitter it contained ∼5 wave cycles rather than 1–2 as in most dolphin signals. We believe that the classification results achieved with this signal could be generalized to signals that are more dolphin-like, as argued in the Discussion.

Echoes from the fish were highly influenced by the angle of acquisition as well as the size, shape and geometry of the swim bladder, internal surface and propagation along different internal pathways within the fish (Figure 3C).

### Simple parametric classification

Because of our limited sample size (three individuals per species), we started off with a simple parametric classification method. To this end, we calculated the echoes' time signal envelopes using the Hilbert transform and extracted the six following parameters from them:

1. The centralized second moment (or variance):
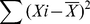
(1)
2. The normalized centralized third moment:
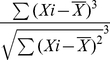
(2)
3. The normalized centralized fourth moment:
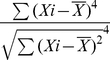
(3)
4. The crest factor (the ratio between the peak amplitude and the root mean square):
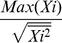
(4)Where 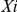 is the amplitude of the echo's time series at sample i, 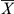 is the average of 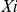.
5. The width (b) and the amplitude (A) of an exponential curve fit to the distribution of the envelopes' amplitudes:
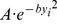
(5)Where 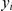 is the probability of finding the amplitude of the echo's time series at the i'th bin of the distribution.

We then used a quadratic discriminant function analysis (DFA) to test classification. We used 90% of the data to train the DFA and 10% to test it and repeated this procedure 10 times (each time with a different 10%/90%) to estimate the standard deviation. This approach is usually termed cross validation. Cross validation was not optimal in the sense that data from the same individual fish (not the same angle) was used to test classification performance, but it was sufficient to assess whether species-specific cues are available in the echoes and if so, lead to the usage of more sophisticated algorithms (SVM, see below). However, to reduce the effect of this undesired procedure we excluded the 4 echoes that were adjacent to the test echoes (2 on each side) from the training (and test) set.

### Classification with Support Vector Machines (SVM)

We used linear Support Vector Machines (SVM, [17], [18]) as the non-parametric classification algorithm. The SVM algorithm was implemented with the “spider” software (http://www.kyb.mpg.de/bs/people/spider).

The main characteristics that make SVMs advantageous for problems of the type we were facing are: 1) Their ability to deal with highly multi dimensional data. 2) The fact that they make no prior assumptions on the data but learn the classification rule from the data itself. For more details about the algorithm and its application to this kind of data see Yovel et al. [13].

A linear SVM can be geometrically described by a separating hyperplane that divides the data into two classes such that the classification error is minimized and the distance from the hyperplane to the closest data points is maximized. The decision rule learned by a linear SVM can be formulated as following:
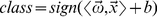
(6)In our case, class is the fish species, 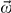 is a vector normal to the hyperplane which is learned by the algorithm, 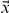 is the data point being classified (a spectrogram of a fish's echo see below) and b is the offset (calculated by the algorithm).

The normal vector 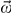 which we will call the ***decision-echo***, can help understanding the features learned by the classification algorithm. When a linear classifier is used (such as in our case) the regions of the decision echo that have high absolute (non zero) values are more important for classification.

As input to the SVMs we used the normalized magnitude of the spectrograms of the echoes in dB. The spectrograms contain both temporal and spectral information, similar to that filtered by the auditory system with the main exception of frequency bands being equally spaced. They do not contain phase information, as is probably the case with the dolphin/porpoise auditory system for such high frequencies.

Because we aimed to test the plausibility of echo based classification and not to compare it to exact behavior or to maximize it, we preferred not to use an auditory model such as a Gamma-tone filter bank. The spectrograms of the echoes were calculated with a Hann window and a 90% overlap between sequential windows. The window length was set to exactly 100 points, therefore providing a 0.1 ms time resolution (smoothed by the window overlap) and a 10 kHz frequency resolution. The spectral resolution is rather low, thus representing a lower boundary of the real resolution possessed by dolphins/porpoises. Temporal resolution is probably plausible for the dolphin/porpoise auditory system [7] and can be reduced by smoothing without harming performance (see below). We preferred to rely on data that is surely available to the animal and not to maximize classification performance. We filtered the spectrogram with a band pass step-function filter cutting out only the frequency range between 60–150 kHz. This helped to get rid of low frequency-noise and made sure that we include only frequencies that are audible to porpoises and dolphins. Finally the spectrograms were transformed to a dB scale and normalized to a maximum of 1. Through the remainder of the text we shall use the term spectrogram to describe the magnitude of the spectrogram.

### Testing classification performance and validation

We tested two types of classification task: 1) The pair-wise situation in which classifiers were trained to classify each species from each of the other two. 2) The one against all situations in which the classifiers were trained to classify one species vs. the other two together.

Classification performance was measured as the total of both types of error (false positive and true negative). The small sample size limited us in two ways: 1) It did not allow us to divide the data into separate training and test sets such assuring that the classifier can apply the rules it learned on new, unseen before, samples of the real world. We were able to partially separate training and test sets as described above for the DFA classifiers. 2) It created a situation in which the dimensionality of the data (determined by the number of pixels in the spectrogram) is of the same order as the number of data points (i.e., echoes) what makes the problem trivial (two 2-dimentional points can always be separated by a line).

We therefore used several validation measures to make sure that our results are not merely an artifact of the small data set and are not purely a result of an over-fitting of the classifier to the specific data set

**Figure 4:**
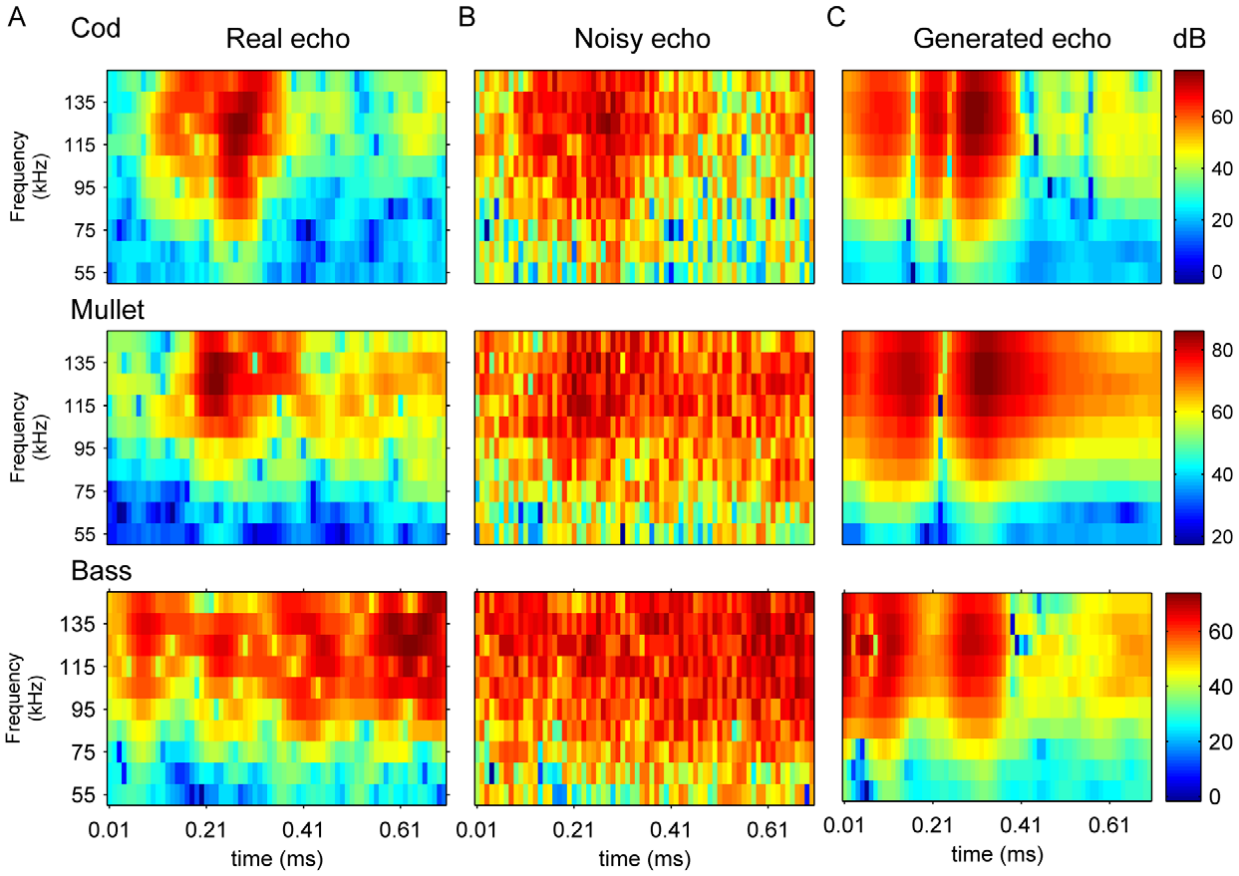
Artificially generated echoes. (A) Original Spectrograms. (B) Noisy spectrograms (same as in A) with 50% noise. (C) Examples for spectrograms generated using the eigen fishes aside original acquired spectrograms. The artificial spectrograms seem simpler (as expected) but they might be very different from the original spectrograms thus increasing the data-set variability.

1. *Smoothing the decision echo:* The decision echo is a weight vectors (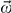) that represents the decision rule learned by the classifier. For a given echo, the species of the fish is determined according to the sign of the inner product of the echo's spectrogram and this weight vector (see Eq. 6 above). To test if the decision rules learned by the classifiers are meaningful, we smoothed the decision echoes with a Gaussian kernel. Smoothing the decision echoes removed high frequency structures that might be an arbitrary artifact of the small sample size. Next, we re-tested classification performance with the new smoothed decision rules that should contain more meaningful features. The decision echoes were smoothed with a 10×10 pixel 2D-Gaussian kernel with a width of σ = 7 pixels normalized to a sum of 1.
2. *Testing sensitivity to noise:* To this end we added Gaussian noise to the spectrograms and tested how does this affect classification performance using classifiers that were trained on the original (‘noiseless’) data set. We tested five increasing noise levels in which the average noise (the mean of the Gaussian) was 10%–50% of the maximum magnitude of the spectrogram (Figure 4B).
3. *Resampling the data using a principle component analysis (PCA):* We used PCA to calculate the principle components (or eigen-vectors) of the spectrograms of each fish species. These vectors (which we shall term eigen-fishes) represent a basis that spans the fish spectrograms in our data set. Each spectrogram is therefore a linear combination of the eigen-fishes of its species. We thus could use the eigen-fishes to generate new spectrograms that are linear combinations of them. We used the first six eigen-vectors per fish to do so because there were three individuals for each species and two aspects (with very different characteristics according to the parametric analysis) for each individual. When generating new spectrograms we made sure that the weights we used were sampled from a distribution much wider than that of the original echoes. This resulted in a new data set that was much more variable than the original one (Figure 4C) and implied that if our classifiers (trained with the original data) are able to classify these new artificial data they might have learned a general classification rule. This third validation method dealt with the two problems mentioned above, namely the number of data points was now ∼5 times larger than the dimensionality of the data and we could separate it into a training set and a test set.
